# Oxygenated hemoglobin as prognostic marker among patients with systemic sclerosis screened for pulmonary hypertension

**DOI:** 10.1038/s41598-023-28608-x

**Published:** 2023-02-01

**Authors:** Panagiota Xanthouli, Ojan Gordjani, Nicola Benjamin, Franziska C. Trudzinski, Benjamin Egenlauf, Satenik Harutyunova, Alberto M. Marra, Nicklas Milde, Christian Nagel, Norbert Blank, Hanns-Martin Lorenz, Ekkehard Grünig, Christina A. Eichstaedt

**Affiliations:** 1 Centre for Pulmonary Hypertension, Thoraxklinik Heidelberg gGmbH at Heidelberg University Hospital, Röntgenstrasse 1, 69126 Heidelberg, Germany; 2 Translational Lung Research Center Heidelberg (TLRC), the German Center for Lung Research (DZL), Heidelberg, Germany; 3 Department of Pneumology and Critical Care Medicine, Thoraxklinik University of Heidelberg, Heidelberg, Germany; 4 Division of Rheumatology, Department of Internal Medicine V: Hematology, Oncology and Rheumatology, University Hospital Heidelberg, Heidelberg, Germany; 5 Department of Translational Medical Sciences, “Federico II” University and School of Medicine, Naples, Italy; 6 Department of Respiratory Care Medicine and Thoracic Surgery, Klinikum Mittelbaden, Baden-Baden Balg, Baden-Baden, Germany; 7 Laboratory for Molecular Genetic Diagnostics, Institute of Human Genetics, Heidelberg University, Heidelberg, Germany

**Keywords:** Biomarkers, Rheumatology, Risk factors

## Abstract

Oxygenated hemoglobin (OxyHem) in arterial blood may reflect disease severity in patients with systemic sclerosis (SSc). The aim of this study was to analyze the predictive value of OxyHem in SSc patients screened for pulmonary hypertension (PH). OxyHem (g/dl) was measured by multiplying the concentration of hemoglobin with fractional oxygen saturation in arterialized capillary blood. Prognostic power was compared with known prognostic parameters in SSc using uni- and multivariable analysis. A total of 280 SSc patients were screened, 267 were included in the analysis. No signs of pulmonary vascular disease were found in 126 patients, while 141 patients presented with mean pulmonary arterial pressure ≥ 21 mmHg. Interstitial lung disease (ILD) was identified in 70 patients. Low OxyHem ≤ 12.5 g/dl at baseline was significantly associated with worse survival (*P* = 0.046). In the multivariable analysis presence of ILD, age ≥ 60 years and diffusion capacity for carbon monoxide (DLCO) ≤ 65% were negatively associated with survival. The combination of low DLCO and low OxyHem at baseline could predict PH at baseline (sensitivity 76.1%). This study detected for the first time OxyHem ≤ 12.5 g/dl as a prognostic predictor in SSc patients. Further studies are needed to confirm these results.

## Introduction

Systemic sclerosis (SSc) is a rare, autoimmune disease characterized by diffuse fibrosis, inflammation and vasculopathy and remains one of the most complex and lethal diseases among connective tissue disorders^1^. The severity of organ involvement predicts the outcome in SSc patients^2,3^.

Pulmonary complications, mainly pulmonary arterial hypertension (PAH) and interstitial lung disease (ILD), remain the leading cause of mortality among SSc patients^1^. Consequently, screening and early diagnosis of both the disease and organ involvement are crucial for prognosis.

The establishment of risk stratification methods is essential to improve the survival through rapid medical and therapeutic interventions. The majority of available scoring models are yet to be validated^1^. Previous studies have already reported the prognostic meaning of impaired oxygen saturation (SaO_2_) during exercise for survival and disease progression among patients with SSc-ILD^4,5^. SaO_2_ may be influenced by both cardiac and pulmonary comorbidities. Furthermore, anemia has been shown to be a prognostic predictor of survival among patients with SSc^6^. A combination of these parameters might therefore be beneficial for risk stratification and prognosis.

Trudzinski et al. showed that apart from white blood cell count, oxygenated hemoglobin (OxyHem), the product of hemoglobin concentration and fractional oxygen saturation (SaO_2_) in arterial capillary blood gas analysis, is a suitable and easy to estimate novel biomarker for the prognosis of patients with chronic obstructive pulmonary disease (COPD)^7^. OxyHem measured in patients with stable COPD, but also during acute COPD exacerbations was independently associated with higher mortality among those patients^8^. OxyHem in arterial blood may also be a novel biomarker for the prognosis in patients with SSc.

Hence, the aim of this study was to analyze the prognostic value of OxyHem in SSc patients referred for screening for pulmonary hypertension (PH). Furthermore, the study investigated the association of OxyHem with the development of pulmonary vascular disease (PVD) among these patients.

## Results

### Baseline clinical and demographic characteristics

**Figure 1:**
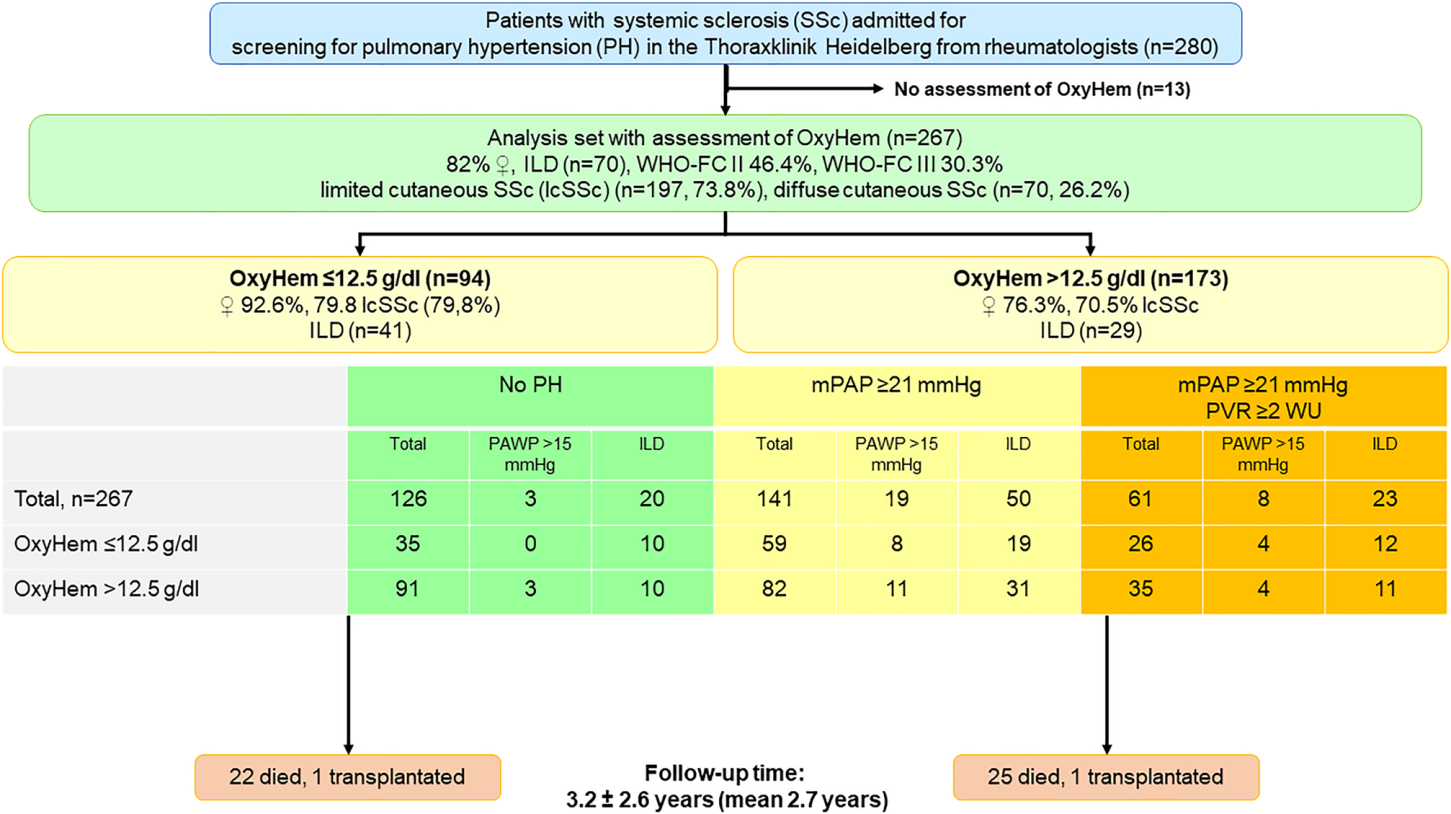
Study flow-chart. The flow-chart of the study cohort gives reasons for exclusion from the analysis set, distribution of patients according to OxyHem and to hemodynamic criteria.

A total of 280 patients with SSc were screened for pulmonary hypertension. From this cohort, 13 patients met exclusion criteria, leading to a final study group of 267 patients (Fig. 1, Table 1). The mean age of the patients was 59.8 ± 13.4 years, 82% were female, 73.8% had limited cutaneous SSc (lcSSc) and 26.2% diffuse cutaneous SSc (dcSSc). The mean duration of SSc at baseline was 9.0 ± 9.3 years. Of these, 70 had significant ILD (26.2%) and 141 (52.8%) showed a mean pulmonary arterial pressure (mPAP) ≥ 21 mmHg (Fig. 1) while 126 had no signs of PH (41.6%). Of the 141 patients with PH 19 presented with PH due to left heart disease (Fig. 1). Patients with PAH received targeted PAH medication according to the contemporary guidelines^9^. Out of 267 patients, 46.4% were in World Health Organization functional class (WHO-FC) II, 30.3% in WHO-FC III and 2.6% in WHO-FC IV. The mean 6-min walking distance (6MWD) was 427.7 ± 102.5 m. Comorbidities were identified in 166 patients: 101 (37.8%) suffered from arterial hypertension, 58 (21.7%) had coronary heart disease, one (0.4%) had reported gastric antral vascular ectasia, three (1%) had lymphoma in the past history and 41 (15.4%) were obese, having a body mass index (BMI) > 30 kg/m^2^.

**Table 1:** Clinical characteristics of the study cohort.

Parameter [unit]	Whole cohort (n = 267)			
	Mean or n ± SD or (%)		95%CI	n
Age [years]	59.78 ± 13.35		58.17 to 61.38	267
Height [cm]	165.47 ± 8.28		164.48 to 166.47	267
Weight [kg]	69.88 ± 15.16		68.05 to 71.70	267
Female sex, no [%]	219	82.0%		267
World Health Organization functional class				267
I	55	20.6%		
II	124	46.4%		
III	81	30.3%		
IV	7	2.6%		
SSc subgroups				267
Limited cutaneous SSc	197	73.8%		
Diffus cutaneous SSc	70	26.2%		
SSc disease duration [years]	9.03 ± 9.31		7.91 to 10.16	265
Modified Rodnan Skin Score	13 ± 11		11 to 14	182
Oxygen saturation [%]	95.84 ± 3.17		95.46 to 96.22	267
Digital ulcers	96	36.8%		261
Arterial hypertension	101	38.0%		266
Interstitial lung disease	70	26.2		267
Hemodynamics at rest				
mPAP [mmHg]	23.01 ± 10.35		21.77 to 24.26	267
PAWP [mm Hg]	10.01 ± 4.51		9.47 to 10.56	266
Cardiac output [l/min]	5.33 ± 1.46		5.15 to 5.51	261
Cardiac index [l/min/m^2^]	3.06 ± 0.78		2.96 to 3.15	264
PVR [WU]	2.68 ± 2.22		2.41 to 2.95	265
Echocardiography at rest				
Right atrial area [cm^2^]	13.3 ± 4.62		12.73 to 13.87	257
Right ventricular area [cm^2^]	15.45 ± 4.18		14.94 to 15.96	260
sPAP [mmHg]	35.38 ± 15.56		33.47 to 37.29	258
TAPSE [mm]	23.46 ± 4.69		22.89 to 24.03	261
Lung function				
VCmax [l]	2.87 ± 0.93		2.76 to 2.99	262
FEV1 [l]	2.27 ± 0.76		2.17 to 2.36	262
TLC [l]	4.98 ± 1.26		4.83 to 5.13	262
DLCO [%]	55.05 ± 18.62		52.73 to 57.41	246
DLCO/VA [%]	69.10 ± 20.99		66.48 to 71.71	250
Laboratory				
NTproBNP [ng/l]	566.33 ± 1297.56		401.34 to 731.33	240
WBC [/nl]	7.73 ± 2.62		7.41 to 8.04	267
Creatinine [mg/dl]	0.88 ± 0.82		0.78 to 0.98	266
GFR [ml/min/1,73m^2^]	84.21 ± 26.47		80.92 to 87.51	250
MCH [pg]	29.73 ± 2.67		29.41 to 30.05	267
MCV [fl]	89.55 ± 6.73		88.74 to 90.36	267
CRP [mg/l]	7.32 ± 10.91		6.01 to 8.63	267
Ferritin [µg/l]	106.97 ± 131.24		87.67 to 126.27	180
Hemoglobin [g/dl]	13.39 ± 1.37		13.22 to 13.55	267
Iron [µmol/l]	13.2 ± 7.99		12.18 to 14.21	241
6-min walking distance [meters]	427.73 ± 102.5		414.72 to 440.73	241

*mPAP* mean pulmonary arterial pressure, *PAWP* pulmonary arterial wedge pressure, *PVR* pulmonary vascular resistance, *WU* Wood Units, *sPAP* systolic pulmonary arterial pressure, *TAPSE* tricuspid annular plane systolic excursion, *VC* vital capacity, *FEV1* forced expiratory volume in first second, *TLC* total lung capacity, *DLCO* diffusion capacity of carbon monoxide, *DLCO/VA* diffusion capacity of carbon monoxide divided by the alveolar volume, *NTproBNP* N-terminal pro-brain natriuretic preptide, *WBC* white blood cells, *GFR* glomerular filtration rate, *MCH* mean corpuscular hemoglobin, *MCV* mean corpuscular volume, *CRP* C-reactive protein.

### Clinical parameters characterizing patients with low OxyHem

The median OxyHem in this cohort was 12.89 g/dl (interquartile range: 151.8). Receiver operating characteristic (ROC) analyses for OxyHem were in line with the cutoff from the literature^7^ of 12.5 g/dl (ROC area under the curve *P* = 0.004). Overall, 94 SSc patients had an OxyHem ≤ 12.5 g/dl and 173 SSc patients OxyHem values above 12.5 g/dl (Table 2). Compared to patients with OxyHem > 12.5 g/dl, patients with low OxyHem were mainly female (n = 87, 92.6% vs. n = 132, 76.3%, *P* = 0.001), had mostly lcSSc (n = 75, 79.8% vs. n = 122, 70.5%, *P* = 0.011), a more impaired iron metabolism in terms of lower ferritin levels (75.3 ± 83.6 vs. 126.7 ± 150.6 µg/l, *P* = 0.004) in normal range, mean corpuscular volume of erythrocytes (MCV) (88.1 ± 8.7 vs. 90.3 ± 5.2 fl, *P* = 0.027) and mean corpuscular hemoglobin in erythrocytes (29.1 ± 3.0 vs. 30.1 ± 2.4 pg, *P* = 0.006). Moreover, they were clinically more limited in terms of WHO-FC (*P* = 0.001), shorter 6MWD (408.6 ± 101.3 vs. 437.8 ± 102.0 m, *P* = 0.036) and greater hemodynamic impairment with a higher pulmonary vascular resistance (PVR) (3.2 ± 2.6 vs. 2.4 ± 1.9 Wood Units (WU), *P* = 0.018) and mPAP (25.6 ± 11.7 vs. 21.6 ± 9.3 mmHg, *P* = 0.005) (Table 2). There was no significant difference in N-terminal pro brain natriuretic peptide (NTproBNP) between the two groups. Out of 94 patients with OxyHem ≤ 12.5 g/dl, 29 suffered from ILD (23.7%). In 59 patients with low OxyHem, mPAP was ≥ 21 mmHg (Fig. 1). Concomitant ILD was diagnosed in 70 patients, out of which 50 showed an mPAP ≥ 21 mmHg. Elevation of pulmonary arterial wedge pressure (PAWP) > 15 mmHg was detected in 22 patients, 19 showed an mPAP ≥ 21 mmHg (Fig. 1). Six patients had PH due to both left heart and lung disease. Pulmonary function test (PFT) parameters were significantly more impaired among patients with lower OxyHem including maximal vital capacity (VCmax), forced expiratory volume in one second (FEV_1_) and total lung capacity (TLC). In the laboratory analysis C-reactive protein (CRP) was higher in the low OxyHem group (*P* = 0.020) and these patients had significantly lower hemoglobin (Hb) (*P* < 0.0001). There was no difference in renal function between the two groups (Table 2).

**Table 2:** Clinical characteristics of the OxyHem subgroups.

Parameter [unit]	Cohort with OxyHem > 12.5 g/dl (n = 173)			Cohort with OxyHem ≤ 12.5 g/dl (n = 94)			*P*
	mean or n ± SD or (%)		n	mean or n ± SD or (%)		n	
Age [years]	58.77 ± 13.4		173	61.63 ± 13.11		94	0.095
Height [cm]	166.66 ± 8.56		173	163.3 ± 7.31		94	0.001
Weight [kg]	71.93 ± 15.27		173	66.1 ± 14.27		94	0.003
Female sex, no [%]	132	76.3%	173	87	92.6%	94	0.001
World Health Organization functional class			173			94	0.001
I	44	25.4%		11	11.7%		
II	83	48.0%		41	43.6%		
III	44	25.5%		37	39.3%		
IV	2	1.2%		5	5.3%		
SSc subgroups			173			94	0.011
Limited cutaneous SSc	122	70.5%		75	79.8%		
Diffus cutaneous SSc	51	29.5%		19	20.2%		
SSc disease duration [years]	8.20 ± 8.99		170	10.55 ± 9.72		94	0.049
Modified Rodnan Skin Score	12 ± 10		120	15 ± 11		62	
Oxygen Saturation [%]	96.41 ± 1.84		173	94.79 ± 4.55		94	0.001
Digital ulcers	56	32.8%	171	40	44.4%	90	
Arterial hypertension	61	35.5%	172	40	42.6%	94	
Interstitial lung disease	41	23.7%	173	29	30.9%	94	0.505
Hemodynamics at rest							
mPAP [mmHg]	21.61 ± 9.26		173	25.6 ± 11.72		94	0.005
PAWP [mm Hg]	9.79 ± 4.32		172	10.42 ± 4.84		94	0.281
Cardiac output [l/min]	5.43 ± 1.59		169	5.15 ± 1.17		92	0.135
Cardiac index [l/min/m^2^]	3.03 ± 0.77		172	3.11 ± 0.8		92	0.425
PVR [WU]	2.42 ± 1.93		172	3.16 ± 2.61		93	0.018
Echocardiography at rest							
Right atrial area [cm^2^]	13.31 ± 4.71		163	13.29 ± 4.49		94	0.967
Right ventricular area [cm^2^]	15.63 ± 4.5		167	15.13 ± 3.52		93	
sPAP [mmHg]	32.88 ± 12.87		164	39.73 ± 18.67		94	
TAPSE [mm]	23.69 ± 4.5		168	23.04 ± 5.01		93	
Lung function							
VCmax [l]	3.04 ± 1.0		170	2.56 ± 0.70		92	< 0.001
FEV1 [l]	2.4 ± 0.80		170	2.02 ± 0.60		92	< 0.001
TLC [l]	5.16 ± 1.29		170	4.64 ± 1.12		92	0.001
DLCO [%]	57.44 ± 18.82		160	50.66 ± 17.5		86	0.006
DLCO/VA [%]	71.89 ± 20.98		162	63.95 ± 20.13		88	0.004
Laboratory							
NTproBNP [ng/l]	478.36 ± 1356.77		154	723.86 ± 1175.35		86	0.160
WBC [/nL]	7.99 ± 2.76		173	7.24 ± 2.26		94	0.025
Creatinine [mg/dl]	0.88 ± 0.94		172	0.89 ± 0.52		94	0.886
GFR [mL/min/1,73m^2^]	85.54 ± 24.42		161	81.82 ± 29.83		89	0.316
MCH [pg]	30.06 ± 2.41		173	29.12 ± 3.02		94	0.006
MCV [fl]	90.32 ± 5.24		173	88.13 ± 8.69		94	0.027
CRP [mg/l]	5.88 ± 6.12		173	9.97 ± 16.13		94	0.020
Ferritin [µg/l]	126.68 ± 150.61		111	75.25 ± 83.58		69	0.004
Hemoglobin [g/dl]	14.11 ± 0.93		173	12.07 ± 1.02		94	< 0.001
Iron [µmol/l]	13.54 ± 5.29		153	12.61 ± 11.25		88	0.465
6-min walking distance [meters]	437.75 ± 102.04		158	408.64 ± 101.26		83	0.036

*mPAP* mean pulmonary arterial pressure, *PAWP* pulmonary arterial wedge pressure, *PVR* pulmonary vascular resistance, *WU* Wood Units, *sPAP* systolic pulmonary arterial pressure, *TAPSE* tricuspid annular plane systolic excursion, *VC* vital capacity, *FEV1* forced expiratory volume in first second, *TLC* total lung capacity, *DLCO* diffusion capacity of carbon monoxide, *DLCO/VA* diffusion capacity of carbon monoxide divided by the alveolar volume, *NTproBNP* N-terminal pro-brain natriuretic preptide, *WBC* white blood cells, *GFR* glomerular filtration rate, *MCH* mean corpuscular hemoglobin, *MCV* mean corpuscular volume, *CRP* C-reactive protein.

### Survival in SSc patients

Within the observation period of 3.2 ± 2.6 (median 2.7; interquartile range 3.5) years 47 patients died (17.6%): 22 due to PH (46.8%), 4 due to PH and/or ILD (8.5%), 3 due to ILD (6.4%), 7 due to cancer (14.9%), one due to pulmonary sepsis (2.1%) and 3 due to left heart disease (6.4%). Two patients underwent lung transplantation due to PH and ILD. In seven cases the cause of death remained unknown (14.9%). The mean estimated overall survival was 7.7 ± 0.3 standard error of the mean years (95% confidence interval 7.1 to 8.2) from baseline. Survival was similar in male and female patients (*P* = 0.624) and between patients with dcSSc and lcSSc (*P* = 0.694).

In the univariable Kaplan–Meier analysis, OxyHem ≤ 12.5 g/dl (*P* = 0.046), age at baseline of ≥ 60 years (*P* = 0.002), the presence of ILD (*P* < 0.0001), high PVR ≥ 2 WU (*P* = 0.002) and low DLCO ≤ 65% predicted (*P* < 0.001) were significantly associated with worse survival (Table 3). White blood cell (WBC) count ≥ 10,000/ml was analyzed together with OxyHem in the initially described COPD cohort^7^. It was also a significant prognostic parameter for survival in our cohort (*P* = 0.02).

**Table 3:** Uni- and multivariable Cox-regression analysis for survival.

Variables	Univariable analysis		Multivariable analysis n = 202
	*P*-value	n	*P*-value
OxyHem ≤ 12.5 g/dl	0.046	256	n.s
WBC ≥ 10,000 /ml	0.020	255	n.s
Known prognostic predictors			
Sex	0.624	256	n.s
Type of SSc	0.694	256	n.s
DLCO ≤ 65% predicted	< 0.001	240	< 0.0001
ILD presence	< 0.0001	256	< 0.0001
Age ≥ 60 years, baseline	0.002	249	0.012
PVR ≥ 2 WU	0.002	253	n.s

*OxyHem* oxygenated hemoglobin, *WBC* white blood cell count, *SSc* systemic sclerosis, *DLCO* diffusion capacity of the lung for carbon monoxide, *ILD* interstitial lung disease, *n.s.* not significant, *PVR* pulmonary vascular resistance, *WU* Wood Units.

**Figure 2:**
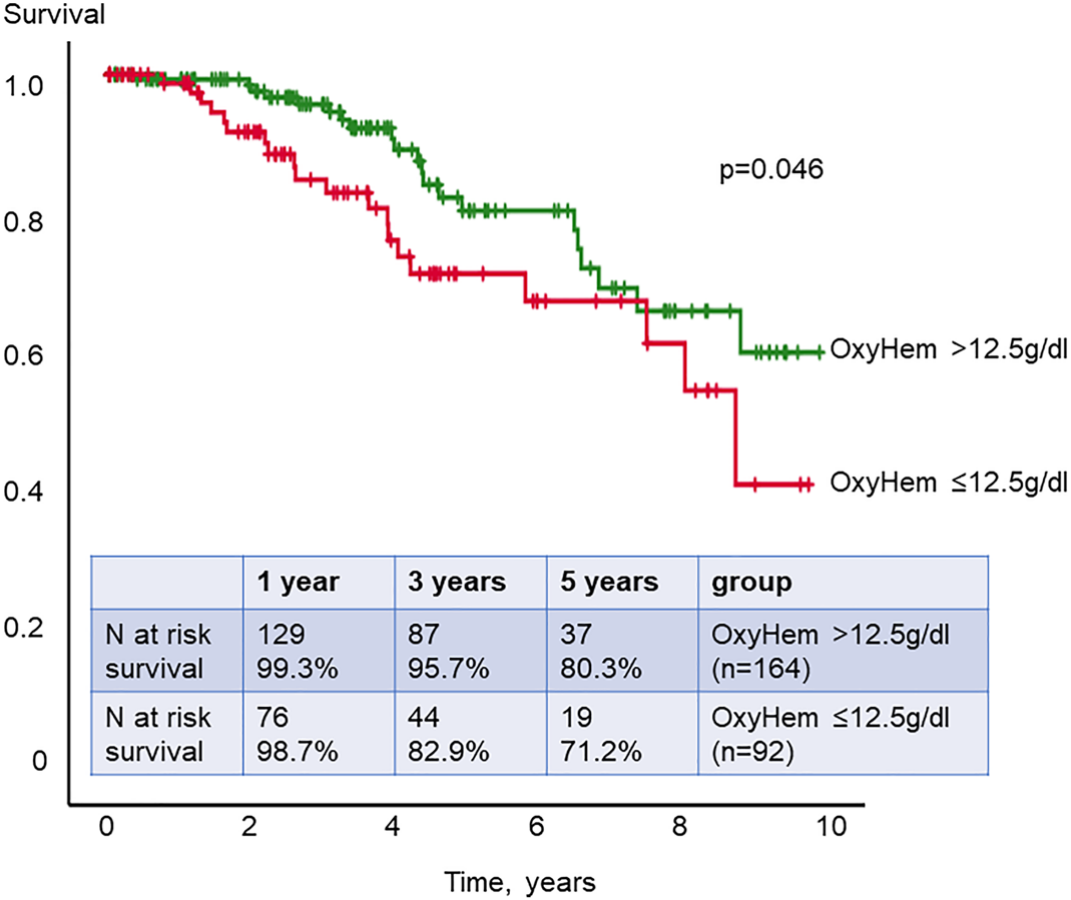
Kaplan–Meier analysis of OxyHem. Patients with an OxyHem value equal and lower than 12.5 g/dl had a significantly shorter survival in our study cohort.

**Figure 3:**
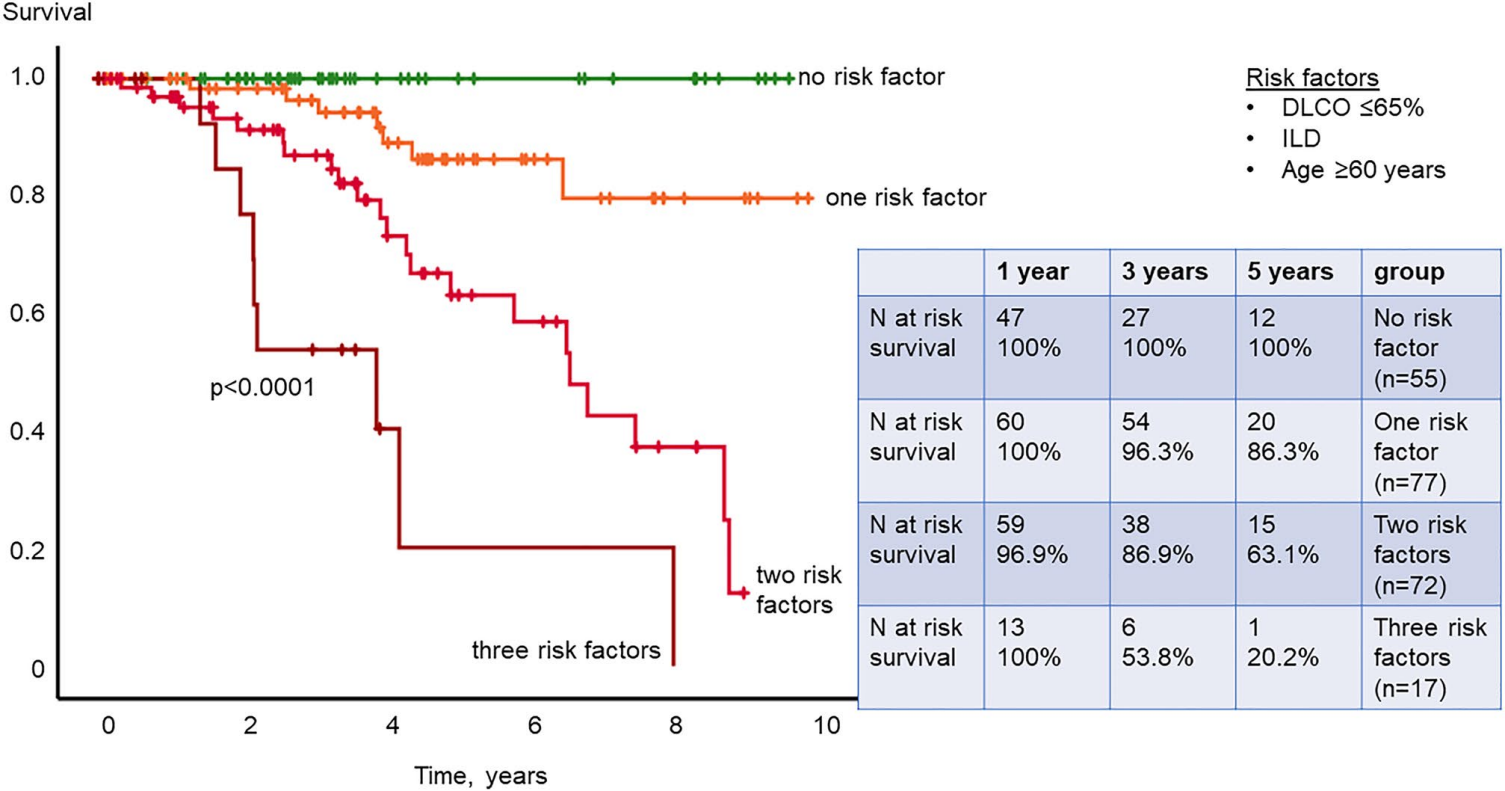
Kaplan–Meier analysis of multivariable risk set. A multivariable risk set including DLCO ≤ 65% predicted, ILD and age ≥ 60 years was used for risk stratification.

Patients with low OxyHem ≤ 12.5 g/dl showed significantly worse survival than patients with OxyHem > 12.5 g/dl with 1-, 3-, 5-year survival of 98.7%, 82.9% and 71.2% versus 99.3%, 95.7% and 80.3%, respectively (Kaplan–Meier *P* = 0.046, Fig. 2). In the multivariable Cox-regression analysis, low DLCO, the presence of ILD and older age were identified as independent prognostic predictors for survival (combined *P* < 0.0001, Fig. 3).

### OxyHem in pulmonary vascular disease

At baseline, patients with PH (mPAP ≥ 21 mmHg) had the highest rate of low OxyHem (41.8%), compared to patients with normal hemodynamics (27.8% low OxyHem; chi-square *P* = 0.016). Low OxyHem at follow-up was though not associated with hemodynamic status (*P* = 0.562) (Table 4). PH at baseline could be predicted with 41.8% sensitivity and 72.7% specificity by low OxyHem. When combining low DLCO and low OxyHem, prediction of PH at baseline had a sensitivity of 76.1% and a specificity of 50.6%. In comparison, step 1 of the DETECT algorithm^10^ reached 85.3% sensitivity and 37.0% specificity. In step 2 of the DETECT algorithm^10^, 79.4% sensitivity and 73.6% specificity could be reached for PH at baseline.

**Table 4:** Frequency of low OxyHem in patients with PH or PAH.

Pulmonary vascular disease	Frequency	Patients not meeting hemodynamic criteria	Chi-square^b^
Pulmonary hypertension mPAP ≥ 21 mmHg	59 out of 141^a^ 41.8%	35 out of 126^a^ 27.8%	*P* = 0.016
Pulmonary arterial hypertension mPAP ≥ 21 mmHg, PVR > 2 WU, PAWP ≤ 15 mmHg	45 out of 99^a^ 45.5%	49 out of 168^a^ 29.2%	*P* = 0.007

^a^The total number refers to the entire cohort meeting the specified hemodynamic criteria in the first column.

^b^Compared to patients not meeting the hemodynamic criteria^29^.

When using the three independent risk factors DLCO ≤ 65% predicted, age ≥ 60 years and presence of ILD, occurrence of PH with mPAP ≥ 21 mmHg during follow-up could be predicted with a sensitivity of 91.1% and a specificity of 34.6% when at least one of the three risk factors was met. The combination of DLCO ≤ 65% predicted and OxyHem ≤ 12.5 g/dl had a sensitivity of 58.7% and a specificity of 72.9% to predict PH during follow-up when at least one of the two risk factors was met (prediction of 44 out of 57; true negative 35 out of 66).

## Discussion

This study detected for the first time that SSc patients with an OxyHem level ≤ 12.5 g/dl have a significantly worse survival than patients with an OxyHem level > 12.5 g/dl (*P* = 0.046). ILD, low DLCO, older age were robust prognostic factors associated with survival in the multivariable analysis. Furthermore, low OxyHem in combination with low DLCO at baseline were predictors of PH at baseline and during follow-up (sensitivity 76.1% and 58.7%, specificity of 50.6% and 72.9%, respectively). To our knowledge, this is the first study indicating the impact of OxyHem as predictor of survival among SSc patients.

### OxyHem and SSc prognosis

Lower OxyHem was identified in patients with a shorter survival, highlighting the impact of oxygenation and the significance of oxygen transport among patients suffering from extended fibrosis and vasculopathy, that typically characterize SSc^11^. Patients with low OxyHem revealed more often clinical characteristics with worse survival in previously described risk stratification models, such as greater WHO-FC^12^, elevated CRP^1^, presence of ILD^1^, lower VCmax and DLCO^13^ as well as lower Hb and shorter 6MWD^12,13^. Patients with lcSSc and female patients had significantly more often a low OxyHem level in our cohort. As these patients would not be suspected to be at risk for an impaired outcome, this underlines the importance of evaluating patients also at low risk^1,12–16^.

### SSc and oxygenation

The importance of oxygen saturation after exercise as a predictor of mortality among SSc-ILD patients was previously evaluated^5^. Exercise induced desaturation was also shown to be predictor of mortality among patients at risk for PH in the PHAROS registry, though SaO_2_ at rest did not differ between the groups^17^ and was found to be associated with 3-year-survival in the DIBOSA study^18^. Wu et al. reported that desaturation < 94% in 6MWD combined with the presence of arthritis was associated with disease progression in SSc-ILD^4^. In contrast, for the calculation of OxyHem, resting oxygen saturation can be used, which makes the parameter easier to determine and less exhausting for the patient compared to assessment of oxygen saturation during exertion.

### PVD

In our cohort, OxyHem was associated with pulmonary vasculopathy only at baseline but not at follow-up. This might be caused by initiation of PAH targeted therapy after diagnosis. Hoeper et al. reported that oxygenation was not a prognostic marker for survival among prevalent patients with idiopathic PAH under therapy, which supports our results^19^. Recently, Valentin et al. (2021) showed that oxygen desaturation (SaO_2_) ≥ 3% at the time of the first follow-up after one year in combination with low DLCO was significantly associated with worse prognosis of PAH^20^, matching our results linking low OxyHem and low DLCO with early PVD. In this cohort, male sex did not correlate with survival consistent with results from the literature^6,21,22^.

### Strengths and limitations of the study

This study provides important insights into prognostic predictors and stratification models in SSc patients screened for PH. The screening for PH was performed in an expert center including a thorough diagnostic workup, leading to a well characterized study cohort. OxyHem offers a parameter, which is pathophysiologically important in different organ involvements in SSc such as ILD, PH and cardiac diseases. While it was a significant predictor in the univariable analysis it was outperformed in the multivariable model. This could be due to the limited sample size or the strong effect of ILD on prognosis. Since we only included extensive ILD in our assessment, this could have also biased the results. One of the main limitations of this study is its retrospective character leading to a smaller data set due to missing parameters. Early prognostic markers need to be investigated in future, prospective studies. We also had no control cohort of healthy individuals or other well characterized patients to compare our results to. However, the prognostic value of OxyHem has already been described in COPD patients^7^. Furthermore, other organ manifestations of SSc, such as gastrointestinal manifestations and renal crisis, could not be considered due to missing data. Although the cohort size of our study is large for such a rare disease, sample size of subgroups was too small to differentiate the impact of OxyHem on survival separately in ILD and PH. Another limitation is that in our cohort blood gas analysis was performed with arterialized capillary blood from the earlobe, which might not be included in standard screening procedures by rheumatologists. However, this method has already shown good correlation with arterial puncture^23^. The meaning of OxyHem during the course of the disease including follow-up data should be addressed in future studies. Finally, our cohort included only SSc patients referred for screening for PH, which could have led to biased data.

### Conclusions

The results of this study revealed that OxyHem may be a new biomarker which could be used for risk stratification in SSc and identification of patients with early PVD and PH, respectively. OxyHem is easy to calculate in daily routine and values ≤ 12.5 g/dl were significantly associated with a worse survival. Moreover, the combination of DLCO ≤ 65% predicted and low OxyHem could predict the development of early PVD in this cohort. Further prospective studies are needed to confirm the results.

## Methods

### Study population

SSc patients with dcSSc or lcSSc ^24^ referred to our center for PH at the Thoraxklinik Heidelberg gGmbH at Heidelberg University Hospital, Germany for screening from their rheumatologists between 2008 and 2020 were retrospectively analyzed in this single center, cross-sectional cohort study. All patients fulfilled the SSc classification criteria of the American College of Rheumatology/European League against Rheumatism^25^ and were followed yearly according to the current recommendations^9^. Individuals were excluded, if they were underaged, had rheumatic diseases other than SSc, had missing data for the calculation of OxyHem or were unable to provide informed consent. Data of part of this cohort has already been analyzed and published before^26^. The ethics committee of the Medical Faculty of Heidelberg University (internal number S-126/2021) had no objection against the conduct of this study. All patients provided written informed consent for the analysis of the data. The study complied with the Declaration of Helsinki in its current version.

### Study design

Data from routinely performed examinations were extracted from the patient's files at the first presentation in the PH center. Patients were referred from their rheumatologists for screening for PH due to unexplained breathlessness and/or suspicion for PH. A detailed clinical work-up was performed in all patients. The routinely performed screening included medical history, recording of comorbidities such as arterial hypertension, coronary heart disease or obesity (BMI > 30 kg/m^2^), physical examination, electrocardiogram, PFT with DLCO, blood gas analysis obtained from arterialized capillary blood from earlobe, echocardiography at rest and during exercise, high resolution computed tomography scan of the lungs (HRCT) to investigate the presence of any pulmonary manifestations or diseases, WHO-FC, 6MWD test under standardized conditions^27^, laboratory examinations including blood count tests, NTproBNP, CRP, analysis for iron deficiency, renal function including glomerular filtration rate, and right heart catheterization at rest and/or during exercise.

The presence of cardiac disease, such as coronary heart disease, and pulmonary manifestations, such as PH or ILD, was documented and, in case of pathology, furtherly examined. ILD was only considered in case of extensive ILD defined as the presence of significant interstitial fibrosis in the HRCT of the lungs (> 20% of the parenchyma) or restrictive pattern in PFT (VCmax < 70% predicted with a normal ratio of FEV1 to VC) in case of missing quantification of interstitial fibrosis in the HRCT^28^. Manifest PAH was diagnosed according to valid guidelines at the time of the study (mPAP ≥ 25 mmHg, PAWP ≤ 15 mmHg and PVR > 3 WU) measured by RHC^9^. Signs of early PVD were defined as mPAP ≥ 21 mmHg. PH due to left heart disease was defined by an mPAP ≥ 21 mmHg with a PAWP > 15 mmHg^29^. PH due to lung disease was based on lung function parameters and in case of ILD HRCT imaging^30^.

### Statistical methods

Statistical analyses were performed by a medical statistician (NB). Data are presented as mean ± standard deviation (SD) with 95% confidence interval of the mean. Frequency data are presented as n and %. The concentration of OxyHem, (g/dl) was calculated at baseline by the multiplication of Hb concentration in blood with the SaO_2_ measured during blood gas analysis as described before^7^.

Survival analysis was performed using the Kaplan-Meier and Cox-regression analysis. The first screening visit date was set as baseline for the survival analysis. Death was defined as death due to any cause. Thresholds for dichotomization of parameters were either obtained by the literature of known prognostic predictors or by ROC in case of unknown threshold or in need of threshold adjustment. Clinical parameters were compared with the two-sided student’s t-test for independent samples for patients above versus below the OxyHem threshold. Qualitative data was analyzed using the chi-square test, respectively. This study is of exploratory nature. P-values < 0.05 were considered as statistically significant and were not adapted for multiple testing.

Variables were evaluated by univariable analysis to identify their impact on survival. Parameters were then analyzed with multivariable Cox-regression analysis. OxyHem was compared to known prognostic factors in SSc, which were identified by literature search. These included sex, type of SSc, age ≥ 60 years at baseline^31^, presence of ILD^1,15^, PVR ≥ 2 WU^26^, DLCO ≤ 65% predicted^13^. In addition, WBC count was included as it showed significant predictive power aside from OxyHem in the OxyHem discovery cohort^7^. For the multivariable Cox-regression all parameters listed above were considered.

Survival analysis was performed in subsets of patients with signs of cardiovascular disease with either increased PVR ≥ 2 WU, or with mPAP ≥ 25 mmHg. All analyses have been performed using IBM SPSS version 27 (SPSS Statistics V27, IBM Corporation, Somers, New York).

### Ethics approval and consent to participate

All data were pseudonymized, and the study was approved by the ethics committee of the medical faculty of Heidelberg University Hospital (internal number S-126/2021). The study complies with the Declaration of Helsinki in its current version. The patients participating in this study provided written informed consent.

## Data Availability

The datasets are available upon reasonable request to the corresponding author.

## Abbreviations

6MWD: 6-Minute walking distance
BMI: Body mass index
CRP: C-reactive protein
dcSSc: Diffuse cutaneous systemic sclerosis
DLCO: Diffusion capacity for carbon monoxide
FEV_1_: Forced expiratory volume in one second
fl: Femtoliters
GFR: Glomerular filtration rate
Hb: Hemoglobin
HRCT: High resolution computed tomography
ILD: Interstitial lung disease
lcSSc: Limited cutaneous systemic sclerosis
MCV: Mean corpuscular volume of erythrocytes
mPAP: Mean pulmonary arterial pressure
NTproBNP: N-terminal pro brain natriuretic peptide
OxyHem: Oxygenated hemoglobin
PAH: Pulmonary arterial hypertension
PAWP: Pulmonary arterial wedge pressure
PFT: Pulmonary function test
PH: Pulmonary hypertension
PVD: Pulmonary vascular disease
PVR: Pulmonary vascular resistance
RHC: Right heart catheterization
ROC: Receiver operating characteristic
SaO_2_: Arterial oxygen saturation
SSc: Systemic sclerosis
SD: Standard deviation
TLC: Total lung capacity
VCmax: Maximal vital capacity
VC: Vital capacity
WHO-FC: World Health Organization functional class
